# Evaluation of oil accumulation and biodiesel property of *Lindera glauca* fruits among different germplasms and revelation of high oil producing mechanism for developing biodiesel

**DOI:** 10.1186/s13068-023-02265-8

**Published:** 2023-01-25

**Authors:** Zixin Lin, Feng Chen, Hongjuan Wang, Jinhe Hu, Lingling Shi, Zhixiang Zhang, Yu Xiu, Shanzhi Lin

**Affiliations:** 1 Beijing Advanced Innovation Center for Tree Breeding By Molecular Design, College of Biological Sciences and Biotechnology, School of Soil and Water Conservation, National Engineering Laboratory for Tree Breeding, Key Laboratory of Genetics and Breeding in Forest Trees and Ornamental Plants, Ministry of Education, Tree and Ornamental Plant Breeding and Biotechnology Laboratory of National Forestry and Grassland Administration, Beijing Forestry University, Beijing, 100083 China; 2 Department of Biochemistry and Molecular Biology, Yanjing Medical College, Capital Medical University, Beijing, 101300 China

**Keywords:** Woody biodiesel, Fuel properties, Oil accumulation, Transcriptional regulation, Provenances, *Lindera glauca* fruit

## Abstract

**Background:**

*Lindera glauca* with rich resource and fruit oil has emerged as novel source of biodiesel in China, but different germplasms show a variation for fruit oil content and FA profile. To develop *L. glauca* fruit oils as biodiesel, a concurrent exploration of oil content, FA composition, biodiesel yield, fuel property and prediction model construction was conducted on the fruits from 8 plus germplasms to select superior genotype for ideal biodiesel production. Another vital focus was to highlight mechanism that govern the differences in oil content and FA profile of different germplasms. The cross-accessions comparisons associated with oil-synthesized gene transcriptional level and oil accumulative amount led to the identification of potential determinants (enzymes, transporters or transcription factors) and regulatory mechanisms responsible for high-quality oil accumulation.

**Results:**

To select superior germplasm and unravel regulatory mechanism of high oil production for developing *L. glauca* fruit oils as biodiesel, 8 plus trees (accession LG01/02/03/04/05/06/07/08) with high-yield fruits were selected to evaluate the differences in oil content, FA profile, biodiesel yield and fuel property, and to construct fuel property prediction model, revealing a variation in the levels of fruit oil (45.12–60.95%), monounsaturated FA (52.43–78.46%) and polyunsaturated FA (17.69–38.73%), and biodiesel yield (80.12–98.71%) across different accessions. Of note, LG06 had a maximum yield of oil (60.95%) and biodiesel (98.71%), and ideal proportions of C18:1 (77.89%), C18:2 (14.16%) and C18:3 (1.55%), indicating that fruit oils from accession LG06 was the most suitable for high-quality biodiesel production. To highlight molecular mechanism that govern such differences in oil content and FA composition of different accessions, the quantitative relationship between oil-synthesized gene transcription and oil accumulative amount were conducted on different accessions to identify some vital determinants (enzymes, transporters or transcription factors) with a model of carbon metabolic regulatory for high-quality oil accumulation by an integrated analysis of our recent transcriptome data and qRT-PCR detection. Our findings may present strategies for developing *L. glauca* fruit oils as biodiesel feedstock and engineering its oil accumulation.

**Conclusions:**

This is the first report on the cross-accessions evaluations of *L. glauca* fruit oils to determine ideal accession for producing ideal biodiesel, and the associations of oil accumulative amount with oil-synthesized gene transcription was performed to identify some crucial determinants (enzymes, transporters or transcription factors) with metabolic regulation model established for governing high oil production. Our finding may provide molecular basis for new strategies of developing biodiesel resource and engineering oil accumulation.

**Supplementary Information:**

The online version contains supplementary material available at 10.1186/s13068-023-02265-8.

## Background

The global population is expected to hit 9.8 billion by 2050, at which the reserved fossil energy (oil and gas) is expected to run out [1, 2]. Thus, exploiting renewable and clean energy source has become an imperative for sustainable economic development and global energy security. Biodiesel, known as FA methyl ester (FAME), is gradually gaining global attention as ecofriendly fuel for its nontoxicity and biodegradability [3], which has been widely used in several countries such as USA, Malaysia, Indonesia, Brazil, Netherlands, Germany and France [4]. In recent years, the Chinese government has designed a series of planning projects to support the development of renewable energy sources, and the oils from some woody plants (such as *Prunus sibirica*, *Pistacia chinensis*, *Xanthoceras sorbifolia* and *Jatropha curcas*) have been utilized as raw material for biodiesel production [5–8].

*Lindera glauca*, small tree species of the Lauraceae family, is widely distributed in the mountainous and lowland districts of China [9–12]. There are many germplasms of *L. glauca* with different oil content and FA compositions in China. Based on our previous studies on 102 fruit samples of *L. glauca* from 9 geographical provenances, some accessions have been identified with rich oil content and high proportion of oleic and linoleic acid, and importantly, the oil content (42.0–53.0%) of ripen fruits [9, 12] was higher than that of traditional woody oil plants [7, 13–15]. All these indicated that *L. glauca* fruit oils may be as potential source of biodiesel feedstock in China. Given the differences in fruit oil content and its FA compositions of different germplasms, it is vital to select superior germplasm with high-quality fruit oil for better development of woody biodiesel. However, an interesting challenge is the mechanism that governed such differences in oil content and FA composition of *L. glauca* fruits across different accessions. Therefore, understanding molecular basis of high oil production in *L. glauca* fruits has become another one imperative for developing woody biodiesel.

Plant oils are an important natural resource of carbon and energy to meet the increasing demands of food, biofuel, and industrial application [16]. In general, carbon source for oil synthesis of oil plants mostly as sucrose from photosynthetic tissues, which in non-green tissues (developing fruits and seeds) is mainly converted to pyruvate (PYR) via glycolysis or to glyceraldehyde 3-phosphate (GAP) via pentose phosphate pathway (PPP) in both cytosol and plastid, as crucial precursor for acetyl-CoA formation destined to de novo FA synthesis [12, 17]. It is known that oil biosynthetic process is composed of FA synthesis and triacylglycerol (TAG) assembly involved in several regulatory enzymes [16], of which d*e novo* FA synthesis is initiated by carboxylation of acetyl-CoA to malonyl-CoA in plastid, and transferred to malonyl-ACP as two-carbon unit for FA elongation via a condensation cycle to elongate acyl chain by two-carbon units. The newly synthesized free FAs are exported from plastid into cytosolic acyl-CoA and incorporated into phosphatidylcholine (PC) for desaturation in endoplasmic reticulum (ER). The traditional pathway to assemble FAs into triacylglycerol (TAG) is via three sequential acylation of G3P with acyl-CoA to produce diacylglycerol (DAG) and subsequent TAG (known as Kennedy pathway) [18]. It is also noted that in oil plants, three potential mechanisms have shown to control FA flux via PC for TAG assembly, including esterification of nascent FA to PC, conversion of FA from PC to DAG for TAG assembly by PDAT, and direct utilization of PC-derived DAG as substrate for TAG biosynthesis by DGAT [16, 18]. All these revealed one complex metabolic regulatory network for oil synthesis at subcellular level. Yet, the mechanism of how carbon is channeled to FA synthesis and how they are assembled into TAG destined for oil accumulation in oil plants is still unclear. Recently, we performed transcriptome sequencing of developing *L. glauca* fruits to identify some enzymes and transcription factors relevant for oil biosynthesis [12], which could help us to gain new insight into the mechanism for controlling differences in oil content and FA composition among different germplasms.

The aim of this sequential study was to select ideal germplasm and to unravel regulatory mechanism of high oil production for better development of *L. glauca* fruit oil as potential feedstock for biodiesel. To this end, 8 selected plus trees (accessions LG01, LG02, LG03, LG04, LG05, LG06, LG07 and LG08) with high-yield fruits was used as material to assess the differences in oil accumulation (content and FA composition), biodiesel yield and fuel properties of fruit oils from all accessions. Also, a triangular model was constructed for biodiesel property prediction of raw fruit oils from different accessions. Such evaluation could help to select superior accession for developing biodiesel. Another focus of this work was to highlight mechanism that govern differences in fruit oil content and FA profile of different accessions, and to gain valuable information for engineering oil accumulation or molecular-assisted selection. In this regard, based on our recent transcriptomic result of developing *L. glauca* fruits, the comparative analysis of cross-accessions correlation of gene transcriptional level by qRT-PCR with oil accumulative amount was performed as an important attempt to identify some crucial regulators (transporters, enzymes and transcription factors) involved specifically in carbon source supply (glycolysis, PPP and acetyl-CoA formation) and oil synthetic process (FA biosynthesis and TAG assembly), with the aim of deriving a central metabolic regulation model for high-quality oil accumulation in *L. glauca* fruits. This study presents for the first time the application of an integrated evaluation of oil content and biodiesel fuel property and analysis of oil accumulative pattern and oil-synthesized gene transcription cross different accessions of *L. glauca* to highlight high oil producing mechanism for developing biodiesel, which could facilitate the development of *L. glauca* fruit oils as biodiesel feedstock, and help to gain new insight into molecular regulatory mechanism of high-quality oil production for engineering oil accumulation in oilseed plants.

## Results

### Variability in fruit oil content and biodiesel yield of 8 selected accessions

**Fig. 1:**
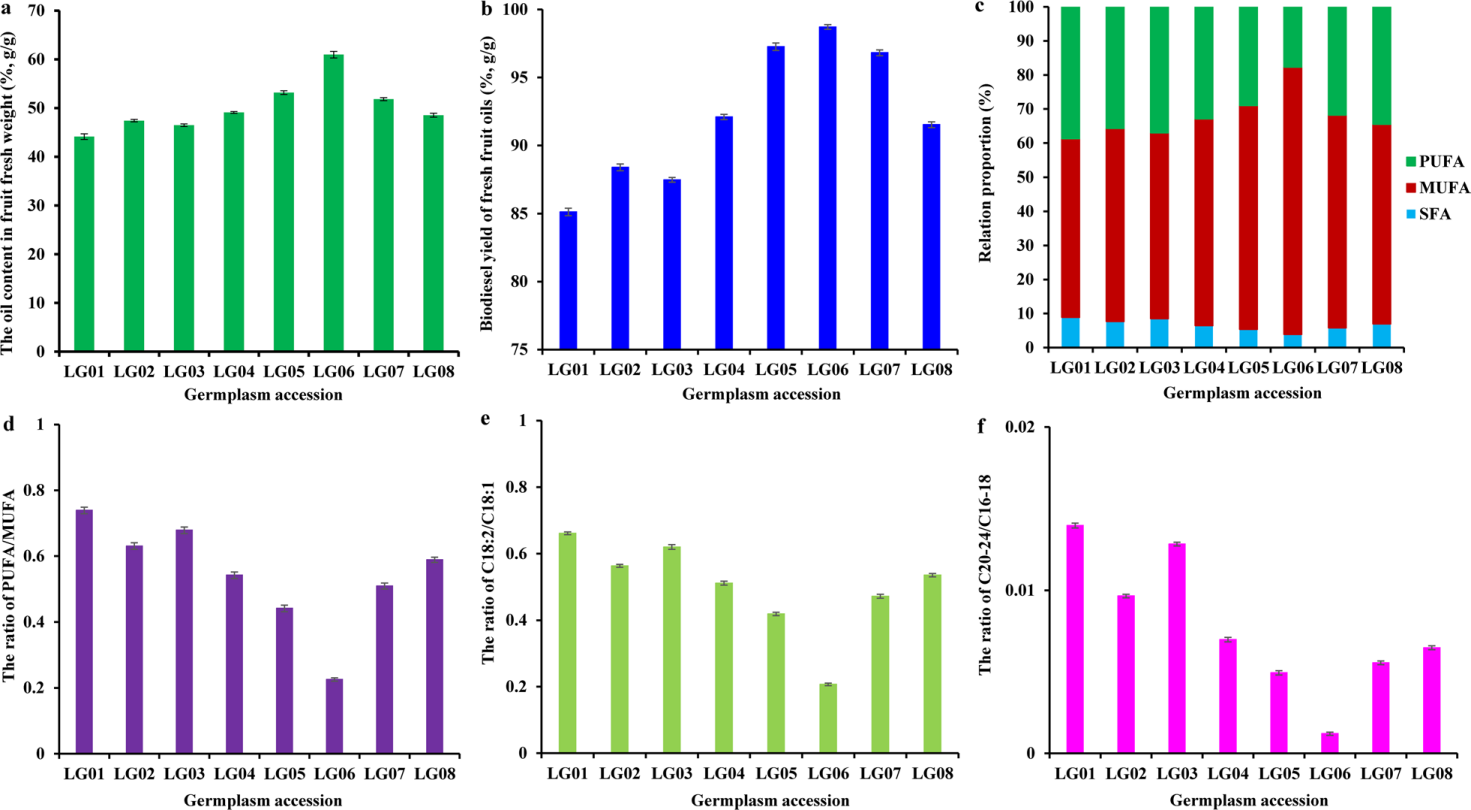
Variabilities on oil content, FA composition ratio and biodiesel yield from *L. glauca* fruits among 8 different germplasm accessions. **a** Variability on fruit oil content from different accessions. **b** Variability on biodiesel yield from fresh fruit oils among different accessions. **c** Variability on relative proportion of SFA, MUFA and PUFA in fruit oils from different accessions. **d** Variability of PUFA/MUFA ratio in fruit oils from different accessions. **e** Variability of C18:2/C18:1 ratio in fruit oils from different accessions. **f** Variability of C20-24/C16-18 ratio in fruit oils from different accessions. Error bars are standard deviations (SD) of three biological replicates

To develop *L. glauca* fruit oils as potential material for biodiesel, it was vital to determine ideal accession with high quantity and quality of fruit oils for gaining maximum economic benefits. Based on our recent studies of different germplasms [9, 12], eight plus trees (germplasm accessions LG01, LG02, LG03, LG04, LG05, LG06, LG07 and LG08) with high fruit yield were selected to investigate the variability in fruit oil content and biodiesel yield of different accessions. Here, the fruit oil contents varied from 44.12% (LG01) to 60.95% (LG06), followed by LG03 (46.48%), LG02 (47.39%), LG08 (48.52%), LG04 (49.09%), LG07 (51.81%) and LG05 (53.16%), of which the fruits of LG05, LG06 and LG07 had oil content more than 51.5% (Fig. 1a). This allowed us to explore the differences in biodiesel yields from fruit oils across all accessions. The biodiesel yield varied among different accessions, ranging from 85.12% (LG01) to 98.71% (LG06) with an average value of 92.17% (Fig. 1b), of which biodiesel yield of LG05 (97.26%), LG06 (98.71%) and LG07 (96.81%) was in the standard of EN 14214 (96.5%). These revealed a difference in fruit oil or biodiesel yield across different accessions, and three high oil-bearing accessions (LG05/06/07) could be valuable as source for developing biodiesel. Yet, the fact of no correlation of oil content with biodiesel yield among different accessions (Fig. 1a, b) indicated that the biodiesel yield may depend on the trans-esterification effectiveness, but not on the oil content of the fruits.

### Variability in FA profiles of fruit oils from 8 selected accessions

Oil content and FA composition are known as two vital factors for determining whether oil plant can be suitable for biodiesel production. Here, 10 kinds of FA compositions were detected in *L. glauca* fruit oils of all accessions (Table 1), including capric acid (C10:0), lauric acid (C12:0), palmitic acid (C16:0), palmitoleic acid (C16:1), stearic acid (C18:0), oleic acid (C18:1), linoleic acid (C18:2), linolenic acid (C18:3), arachidic acid (C20:0) and eicosenoic acid (C20:1). The dominant compound was oleic acid (51.24–77.89%) with an average of 59.52%, followed by linoleic acid (16.14–33.87%), palmitic acid (2.65–5.26%) and linolenic acid (1.55–4.85%), but the other showed minor quantities (0.03–0.46%), of which the maximum value of C18:1 (77.89%) and minimum value of C18:2 (16.14%) and C18:3 (1.55%) were all detected for accession LG06. Also, the total content of C18:1 and C18:2 in fruit oils varied from 84.29% to 94.04%, and three accessions (LG05, LG06 and LG07) had C18:1 content of more than 60% (Table 1), implying that they were ideal raw material for biodiesel.

**Table 1:** Variability on the contents of FA compositions in *L. glauca* fruit oils among 8 different germplasm accessions

Accessions	C10:0 (%)	C12:0 (%)	C16:0 (%)	C16:1 (%)	C18:0 (%)	C18:1 (%)	C18:2 (%)	C18:3 (%)	C20:0 (%)	C20:1 (%)
LG01	0.29 ± 0.03	0.25 ± 0.03	5.26 ± 0.41	1.07 ± 0.08	1.79 ± 0.13	51.24 ± 2.28	33.87 ± 2.01	4.86 ± 0.78	1.25 ± 0.05	0.12 ± 0.03
LG02	0.46 ± 0.03	0.14 ± 0.07	4.64 ± 0.18	1.28 ± 0.24	1.69 ± 0.35	55.14 ± 2.11	31.07 ± 1.45	4.63 ± 0.21	0.74 ± 0.03	0.21 ± 0.05
LG03	0.25 ± 0.03	0.24 ± 0.03	5.13 ± 0.22	2.33 ± 0.14	1.76 ± 0.24	52.04 ± 2.29	32.25 ± 2.05	4.74 ± 0.29	1.11 ± 0.13	0.15 ± 0.03
LG04	0.19 ± 0.04	0.17 ± 0.04	4.04 ± 0.09	2.29 ± 0.21	1.42 ± 0.28	58.30 ± 2.21	29.83 ± 1.21	3.07 ± 0.19	0.61 ± 0.06	0.08 ± 0.02
LG05	0.17 ± 0.03	0.08 ± 0.03	3.54 ± 0.31	2.24 ± 0.17	1.16 ± 0.11	63.32 ± 3.37	26.52 ± 1.21	2.48 ± 0.17	0.38 ± 0.04	0.11 ± 0.04
LG06	0.04 ± 0.02	0.06 ± 0.03	2.65 ± 0.18	0.54 ± 0.13	1.01 ± 0.21	77.89 ± 4.21	16.14 ± 1.28	1.55 ± 0.11	0.09 ± 0.04	0.03 ± 0.04
LG07	0.30 ± 0.11	0.06 ± 0.02	3.85 ± 0.17	0.98 ± 0.21	1.17 ± 0.11	61.28 ± 3.95	28.93 ± 1.45	2.87 ± 0.13	0.41 ± 0.05	0.14 ± 0.34
LG08	0.28 ± 0.09	0.21 ± 0.08	4.41 ± 0.14	1.54 ± 0.31	1.47 ± 0.21	56.98 ± 2.01	30.51 ± 1.43	3.96 ± 0.12	0.58 ± 0.03	0.06 ± 0.02

Error bars are standard deviations (SD) of three biological replicates

The ideal plant oils for high-quality biodiesel production generally contain a small amount of saturated FA (SFA), high amount of monounsaturated FA (MUFA), and low level of polyunsaturated FA (PUFA) [7, 15]. Here, the change analysis for SFA, MUFA and PUFA in fruit oils from different accessions showed that the contents of MUFA and PUFA varied from 52.43% to 78.46%, and from 17.69% to 38.73%, respectively, but the SFA content of all accessions was less than 9% (Fig. 1c). Of note, LG06 had the highest content of MUFA (78.46%) and the lowest amounts of PUFA (17.69%) and SFA (3.85%), revealing that the fruit oils from accession LG06 with ideal FA compositions could meet the demand of high-quantity biodiesel production.

Also of note was low ratio of PUFA/MUFA (especially C18:2/C18:1) or C20-24/C16-18 as key parameter for tribological property of plant oils for industrial application [19]. Here, the lowest ratios of PUFA/MUFA (0.225), C18:2/C18:1 (0.207) and C20-24/C16-18 (0.001) were noted for accession LG06, but the other accessions had a relative high ratios of PUFA/MUFA (0.442–0.739), C18:2/C18:1 (0.419–0.661) and C20-24/C16-18 (0.005–0.014) (Fig. 1d–f), implying that the fruit oils from LG-06 may have ideal tribological properties.

These outcomes, together with the highest yield of oil and biodiesel for accession LG06, revealed that its fruit oil was the most suitable for biodiesel production.

### Evaluation of biodiesel fuel properties of fruit oils from different accessions

Effective evaluation biodiesel fuel properties of iodine value (IV), cetane number (CN), oxidation stability (OS), cloud point (CP), cold filter plugging point (CFPP), density (*D*), kinematic viscosity (KV), degree of unsaturation (DU) and chain length saturated factor (LCSF) would provide a vital basis for exploiting biodiesel plants. Given a notable variation on oil content and total proportion of C18:1 and C18:2 in the fruits of different accessions (Table 1), it was important to assess fuel properties of biodiesel derived from fruit oils of different accessions for determining superior accession as biodiesel production.

CN, one key criterion for ignition delay time and combustion quality of biofuels, was detected to be varied from 48.81 to 51.15 in the FAMEs from fruit oils of all accessions, all of which could satisfy the standard of USA (ASTM D6751: CN > 47), and most accessions (except LG01/03) was also in line with China standard (GB/T 20828: CN > 49), while only LG06 (51.15) could meet Europe standard (EN 14214: 51 < CN < 65) (Table 2), implying that the biodiesels from fruit oils of all accessions had good ignition quality. Also, IV is a crucial index for assessing the unsaturated degree (DU) of FA and OS of biodiesel [20]. The IV values of all accessions varied from 102.15 to 115.09 (Table 2), all of which was less than the specified maximum limit (120) of EN 14214 standard.

**Table 2:** Evaluations of biodiesel fuel properties of fruit oils of *L. glauca* from 8 different germplasm accessions

Accessions or standards	Biodiesel fuel properties^a^									
	DU	LCSF	CN	IV (g/100 g)	CFPP (°C)	CP (°C)	OS (h)	KV (mm^2^ s^−1^, 40 °C)	D (kg/m^−3^, 15 °C)	Methyl esters yield (%)^b^
Accessions										
LG01	134.75 ± 2.15	2.87 ± 0.32	48.81 ± 1.14	115.09 ± 1.7	− 9.74 ± 0.14	− 7.11 ± 0.08	2.59 ± 0.03	4.38 ± 0.14	887.14 ± 1.18	85.12 ± 0.27
LG02	132.66 ± 1.64	2.37 ± 0.24	49.06 ± 1.25	113.69 ± 1.4	− 10.61 ± 0.15	− 7.89 ± 0.07	2.68 ± 0.02	4.44 ± 0.08	878.23 ± 1.74	88.39 ± 0.25
LG03	133.24 ± 2.23	2.75 ± 0.18	48.98 ± 0.96	114.08 ± 1.2	− 9.94 ± 0.13	− 7.30 ± 0.07	2.65 ± 0.01	4.55 ± 0.23	883.34 ± 1.65	87.48 ± 0.18
LG04	129.54 ± 1.84	2.06 ± 0.101	49.43 ± 0.87	111.61 ± 1.5	− 11.16 ± 0.13	− 8.49 ± 0.10	2.79 ± 0.02	4.68 ± 0.19	877.28 ± 1.53	92.09 ± 0.21
Lg05	126.15 ± 1.65	1.70 ± 0.12	49.84 ± 0.68	109.34 ± 1.2	− 11.79 ± 0.16	− 9.12 ± 0.09	2.93 ± 0.02	4.36 ± 0.07	876.35 ± 1.09	97.26 ± 0.23
LG06	115.39 ± 1.24	1.24 ± 0.09	51.15 ± 1.01	102.15 ± 0.9	− 12.59 ± 0.15	− 9.91 ± 0.09	3.34 ± 0.03	4.31 ± 0.06	871.54 ± 0.94	98.71 ± 0.25
LG07	128.87 ± 1.91	1.79 ± 0.12	49.52 ± 0.93	111.16 ± 1.1	− 11.63 ± 0.11	− 8.96 ± 0.09	2.82 ± 0.01	4.48 ± 0.11	876.09 ± 1.09	96.81 ± 0.19
LG08	131.48 ± 1.72	2.17 ± 0.14	49.20 ± 0.79	112.90 ± 1.3	− 10.97 ± 0.09	− 8.31 ± 0.08	2.71 ± 0.02	4.58 ± 0.12	885.13 ± 1.91	91.52 ± 0.16
Standards										
ASTM D6751	–	–	> 47	–	–	− 12∼− 3	≥ 3	1.9∼6.0	–	–
EN 14,214	–	–	> 51	≤ 120	< 5	–	≥ 6	3.5~5.0	860∼900	96.5
GB/T 20,828	–	–	> 49	–	Report	–	≥ 6	1.9∼6.0	820∼900	–

^a^*DU* degree of unsaturation, *LCSF* chain length saturated factor, *CN* cetane number, *IV* iodine value, *CFPP* cold filter plugging point, *CP* cloud point, *OS* oxidation stability, *KV* kinematic viscosity, *D* density. Error bars are standard deviations (SD) of three biological replicates

^b^The biodiesel yield was expressed as the percentage (%, g/g) of the obtained total amount of FA methyl esters (g) to the used amount of raw oils (g)

KV is one key parameter for defining flow capability of biodiesel, used for estimating spray penetration and atomization of fuel [20]. The range of KV value (4.31–4.68 kg/m^3^) of biodiesels from all accessions could meet with the standards of ASTM D6751 (1.9 < KV < 6.0), EN 14214 (3.5 < KV < 5.0) and GB/T20828 (1.9 < KV < 6.0) (Table 2), revealing a good flow or spray capability of biodiesel fuel from *L. glauca* fruit oils of all accessions. Also, density (*D*) is one of vital fuel properties for assessing fuel transferred quantity by injection system for combustion [21]. The D value ranged from 871.54 to 887.14 mm/s of the biodiesels (Table 2), which satisfied with the standards of EN 14214 (860 < D < 900) and GB/T20828 (820 < D < 900), and thus concluded that the biodiesels from *L. glauca* fruit oils of all accessions possessed ideal combustion efficiency.

OS, as a crucial parameter, involves in the level and stability of biodiesel reaction with air [20]. In this study, the OS values of biodiesels from fruit oils of all accessions varied from 2.59 to 3.34 h (Table 2), all of which did not reach the minimum limit (6 h) specified in the standards of EN 14214 and GB/T 20828, but only accession LG06 (3.34 h) could meet the ASTM D6751 standard (OS > 3.0 h), which was likely attributed to low content of PUFA (17.69%) in the fruit oils of LG06 compared with other accessions (29.01–38.73%) (Fig. 1c).

CFPP and CP, two vital low-temperature parameters, are used to describe the maximum of filterability, but not limited by the standards of US and European. The CFPP valve ranged from − 12.59 to − 9.74 °C for biodiesels across all accessions (Table 2), less than the maximum limit (0 °C) of Germany standard (DIN V51606) in summer, and this value for most accessions (except LG01/03) was lower than the minimum limit (− 10.0 °C) for spring and autumn, implying a good cold flow performance of biodiesel. Also, CP is one indicator for controlling fuel at low temperature [21], and its value ranged from − 9.91 to − 7.11 °C across all accessions (Table 2), which could meet ASTM D6751 standard (− 12 °C < CP < − 3 °C). This implied a better cold flow property of biodiesel from all accessions, especially LG06 with the minimum value of CFPP (− 12.59 °C) and CP (− 9.91 °C), which may be mostly attributed to high content of unsaturated FA (Fig. 1c) and small amount of length chain saturated factor (LCSF) (Table 2).

DU and LCSF are known as two parameters based on the type of FAs. Of note, the values of CN, IV and OS are determined greatly by the DU [15], but both CFPP and CP mostly depend on LCSF [15, 19]. The DU value varied from 115.39 to 134.75 across all accessions, of which LG-06 had a minimum of 115.39, coincided with the highest values of CN (51.15) and OS (3.34 h) and the lowest IV value (102.15) (Table 2). However, a negative correlation of LCSF with CFPP or CP was recorded across all accessions (Table 2). It seems therefore that both DU and LCSF may be as critical indicators for evaluation of biodiesel fuel properties of fruit oils of *L. glauca* from different accessions.

Another was concerned about the content of C18:3 and the FAs with four double bonds in the FAMEs. Here, low amount (1.55–4.86%) of C18:3 in fruit oils of all accessions and no four double-bond FAs (C18:4 and C20:4) in the FAMEs (Table 2) all satisfied the EN14214-2008 specification (< 12% and 1%, respectively).

### Construction of prediction model for biodiesel properties of raw fruit oils from different accessions

**Fig. 2:**
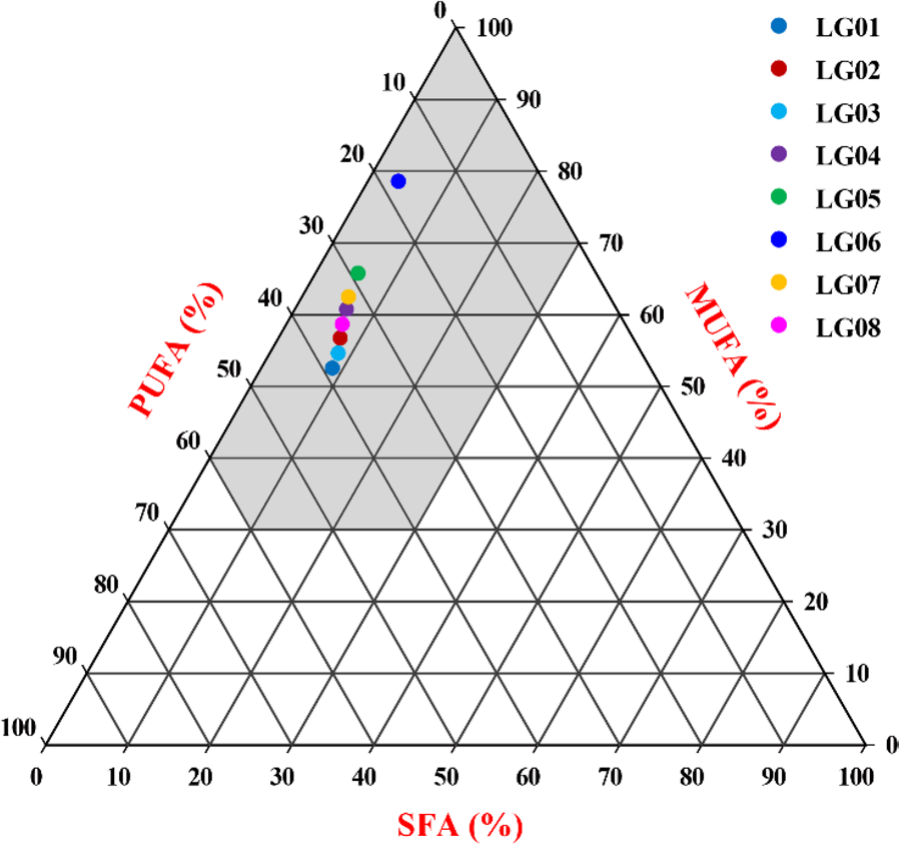
Construction of prediction model for biodiesel fuel properties of raw fruit oils from different accessions. The gray part of region was clearly delineated to predict biodiesel fuel properties that could fully meet the limit of cetane number, iodine number, cold filter plugging point and oxidation stability

Determining biodiesel fuel property is very difficult because it takes a lot of time and cost and thus several attempts have been made to use effective methods to calculate or predict fuel property of biodiesel [15, 22]. Recently, triangular predict model was constructed to effectively evaluate fuel properties of biodiesel based on FA compositions of raw oils from developing *P. sibirica* and *L. glauca* fruits [7, 12]. Such prediction as an attempt was performed here. To this end, the percentages of SFA, MUFA and PUFA in *L. glauca* fruit oils of all accessions (Fig. 1c) were used as three angular points to establish a triangular graph (Fig. 2), in which one specific region (marked in gray) was delineated to predict biodiesel fuel property for fruit oils from all accessions, taking into account the satisfactions of key fuel properties (CN, IN, CFPP, OS and CP). All accessions were presented in the gray area of our constructed triangular graph (Fig. 2), of which accession LG06 was located at the far end of PMFA angular point (lower left vertex) and SFA angular point (lower right vertex), indicating that the fruit oils from all accessions (especially LG06) may be as potential material for biodiesel, coincided with our evaluated results of fuel properties for different accessions (Table 2). Thus, triangular predict model for fuel properties by FA compositions of raw oils could provide simple and effective selection of ideal plant resource biodiesel.

Together, the accession (LG05/06/07) with high fruit oil content and biodiesel yield, and ideal fuel property, could be suitable for high-quality biodiesel production. Yet, another question was the mechanism that governed such difference in oil content and FA composition of *L. glauca* fruits across all accessions (Fig. 1a and Table 1). Effective increases in oil content and ideal FA profile require to unravel complex metabolic regulation network. Thus, in the following, our work focused on the identification of key regulators (enzymes, transcription factors and transporters) specific for carbon source supply and oil synthetic process (FA synthesis and TAG assembly) by the comparative analysis of cross-accessions association of fruit oil content with gene transcript level. Such exploration could gain a better insight into molecular regulation mechanism governing high oil production and provide new biotechnological targets to improve oil yield.

### Transcript differences of carbon allocation specific for acetyl-CoA generation in fruits of different accessions

**Fig. 3:**
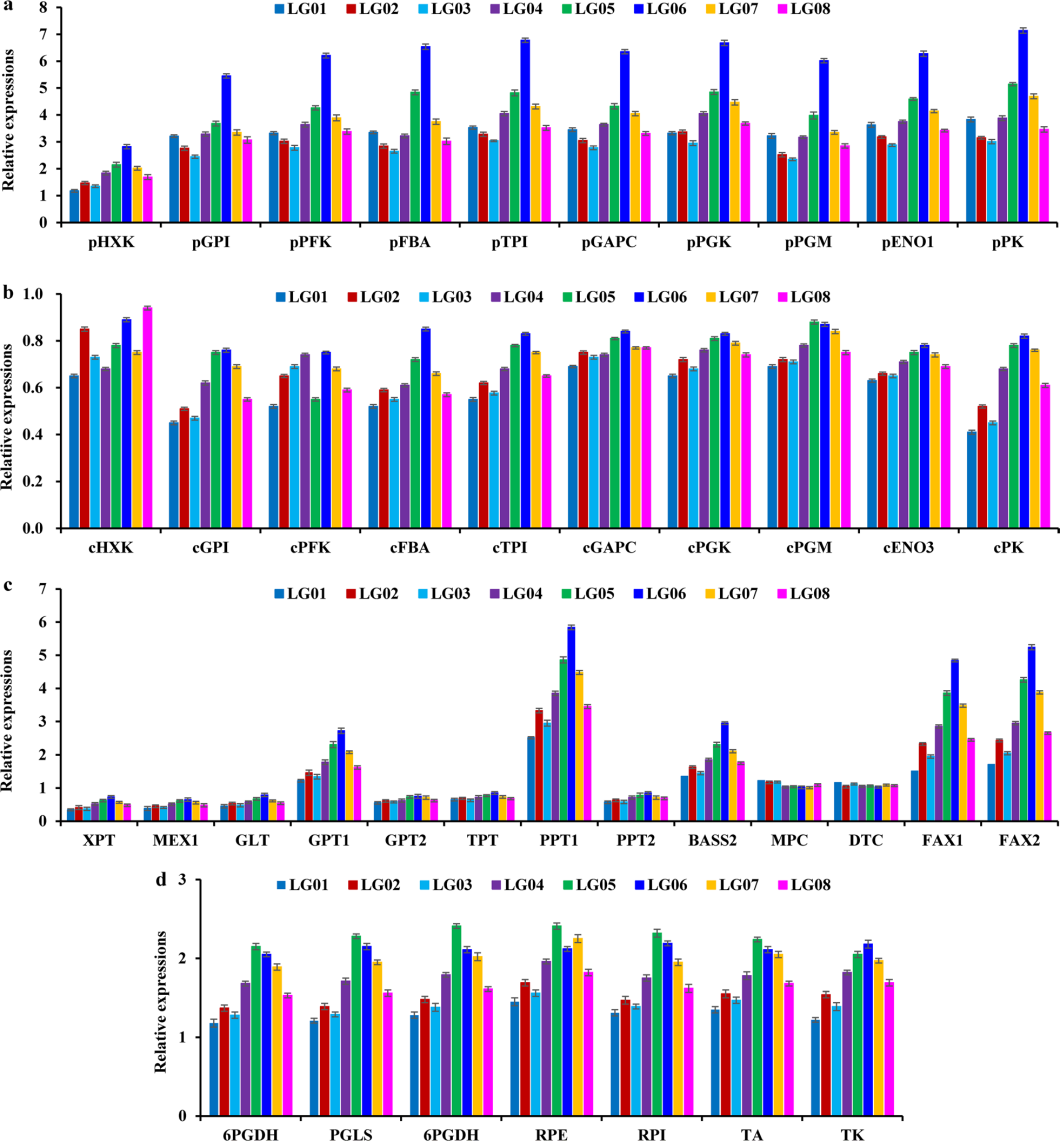
Transcript assay of enzymes and transporters for carbon allocation destined to acetyl-CoA formation in *L. glauca* fruits of different accessions by qRT-PCR. **a** Transcript differences for enzymes involved in plastidic glycolysis among different accessions. **b** Transcript differences for enzymes involved in cytosolic glycolysis among different accessions. **c** Transcript differences for plastidic and mitochondrial transporters involved in transport of various intermediates. **d** Transcript differences for enzymes involved in plastidic PPP among different accessions. The genes for ubiquitin-conjugating enzyme (*UBC*) and large subunit ribosomal protein L32e (*RPL32e*) were used as internal controls, and its expression level was arbitrarily set to 1.00 for standardization. The plastidic (p) and cytosolic (c) isoforms of enzymes are indicated by a prefix in figure (a) and (b), respectively. Error bars are SD of three biological replicates with three technical repetitions each

In oil plants, acetyl-CoA, one vital precursor for FA synthesis, is mainly derived from PYR via glycolysis or GAP via PPP in both cytosol and plastid by a series of regulatory enzymes [17, 23, 24]. Recently, our transcriptomic assay showed that oil accumulation of developing *L. glauca* fruits was regulated by differential transcripts of enzymes between plastidic and cytosolic glycolysis [12], including ATP-dependent phosphofructokinase (PFK), hexokinase (HXK), fructose-bisphosphate aldolase (FBA), phosphoglycerate kinase (PGK), triosephosphate isomerase (TPI), enolase (ENO), GAP dehydrogenase (GAPC) and pyruvate kinase (PK). To determine the relative flux of PYR from plastid or cytosol glycolysis destined to oil production in the fruits of different accessions, transcript differences of all enzymes of two glycolytic pathways were analyzed in the fruits of all accessions by qRT-PCR. The transcript levels of plastid glycolytic enzymes (HXK, PFK, FBA, TPI, GPI, GAPC, PGK, PGM, ENO1 and PK) increased with the increasing amount of fruit oils across all accessions, of which the richest transcripts were all noted for accession LG06 with the highest oil content (Figs. 1a, 3a). Yet, all cytosolic isoforms showed less transcript across all accessions (Fig. 3b). All these revealed a major role of plastid glycolysis in supply PYR for FA synthesis in the fruits of all accessions. Another note with regard to glycolysis was about transporter in interchange of glycolytic intermediate between cytosol and plastid [7, 25]. Given that the orthologs for triose phosphate transporter (TPT), G6P transporter (GPT1/2), phosphoenolpyruvate transporter (PPT1/2), xylulose 5-phosphate transporter (XPT), glycolipid transporter (GLT), and bile acid/sodium symporter (BASS2) was marked in developing *L. glauca* fruits by recent transcriptome analysis [12], it was crucial to explore which of them may contribute to transport glycolytic metabolite from cytosol into plastid for fruit FA synthesis of different accessions. Only GPT1, PPT1 and BASS2 showed high transcript and a high correlation to the amount of fruit oils across all accessions (Figs. 1a, 3c), indicating that GPT1/PPT1/BASS2 may contribute to allocate cytosolic glycolytic metabolites (G6P, PEP or PYR) into plastid for FA synthesis destined to the eventual oil production in *L. glauca* fruits of all accessions.

Aside from glycolysis, FA biosynthesis was fed by GAP via PPP [7, 26]. Our recent annotation of a complete plastidic PPP with differential transcripts in developing *L. glauca* fruits by transcriptome assay [12], allowed to address whether the fruit oil contents of different accessions were correlated to the increasing number of their transcripts. All enzymes of plastidic PPP, including 6-phosphogluconate dehydrogenase (6PGDH), transaldolase (TA), 6-phosphogluconolactonase (PGLS), G6P dehydrogenase (G6PDH), ribulose-5-phosphate (RP) epimerase (RPE), RP isomerase (RPI) and transketolase (TK), were detected with the abundantly coordinated transcripts of all accessions by qRT-PCR (Fig. 3d), and their transcript levels were associated with the amount of fruit oils across all accessions (Figs. 1a, 3d), pointing to a role of plastidic PPP in provision of GAP for FA synthesis.

**Fig. 4:**
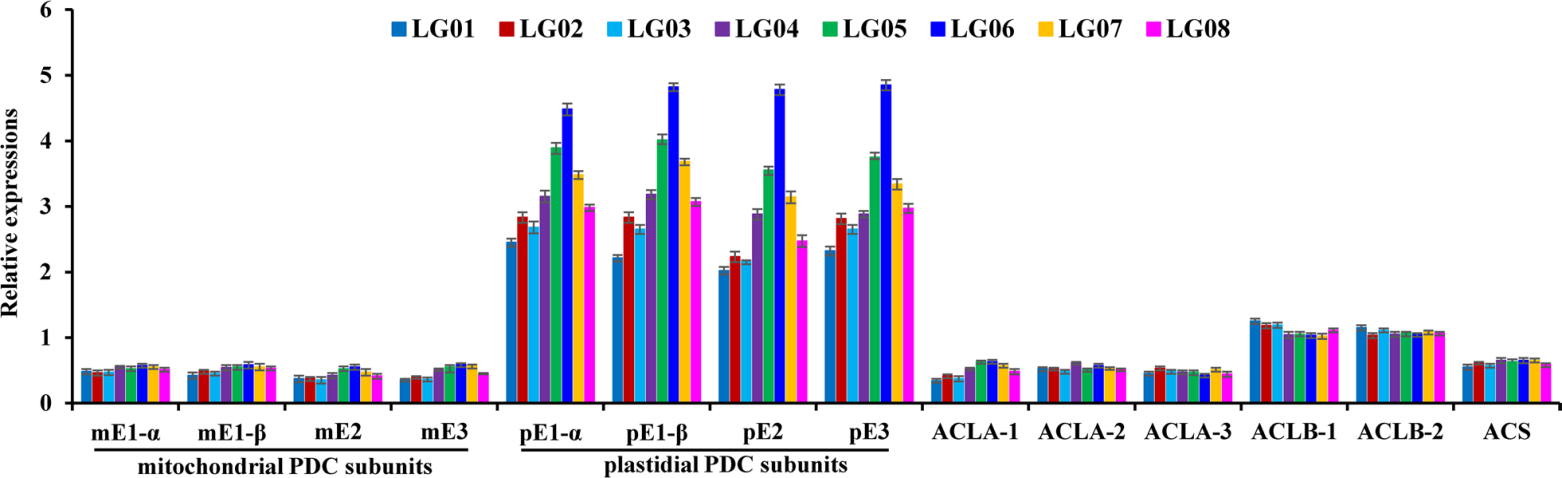
Assay of transcript differences in alternative enzymes for acetyl-CoA generation in *L. glauca* fruits of different accessions by qRT-PCR. Both *RPL32e* and *UBC* genes were used as internal controls, and expression level of inner reference gene was arbitrarily set to 1.00 for standardization. Error bars are SD of three biological replicates with three technical repetitions each

Another was concerned about acetyl-CoA generation from PYR via acetyl-CoA synthetase (ACS), ATP-citrate lyase (ACL), or PYR dehydrogenase complex (PDC) [24], all of which were detected with differential transcripts in developing *L. glauca* fruits by our recent transcriptome assay. Hence, we performed the cross-accessions comparisons of transcripts to explore which of them may specifically devote to allocate PYR flux for acetyl-CoA formation destined for FA synthesis in all accession fruits. High transcript of plastidic PDC was closely consistent with the increase of fruit oil content across the accessions (Figs. 1a, 4), while transcript level of cytosolic ACLB subunits (ACLB-1/-2) showed no notable up-regulation, and less transcript was detected for mitochondrial PDC, cytosolic ACLA and plastidic ACS (Fig. 4), implying that plastid PDC may mostly contribute to acetyl-CoA formation for FA synthesis in all accession fruits. Also of note was the roles of cytosolic ACLB and mitochondrial PYR carrier (MPC), citrate synthase (CS) and dicarboxylate/tricarboxylate carrier (DTC) in cytosolic acetyl-CoA generation for FA elongation [7]. Here, low transcript of mitochondrial MPC, CS4 and DTC was similar to that of cytosolic ACLB and mitochondrial PDC (Figs. 3c, 4), corresponding to small amount of C20:0 and C20:1 in fruit oils (Table 1), pointing to a role of them in providing cytosolic acetyl-CoA for FA elongation.

### Transcript differences of enzymes and transporters for FA and TAG synthesis in fruits of different accessions

**Fig. 5:**
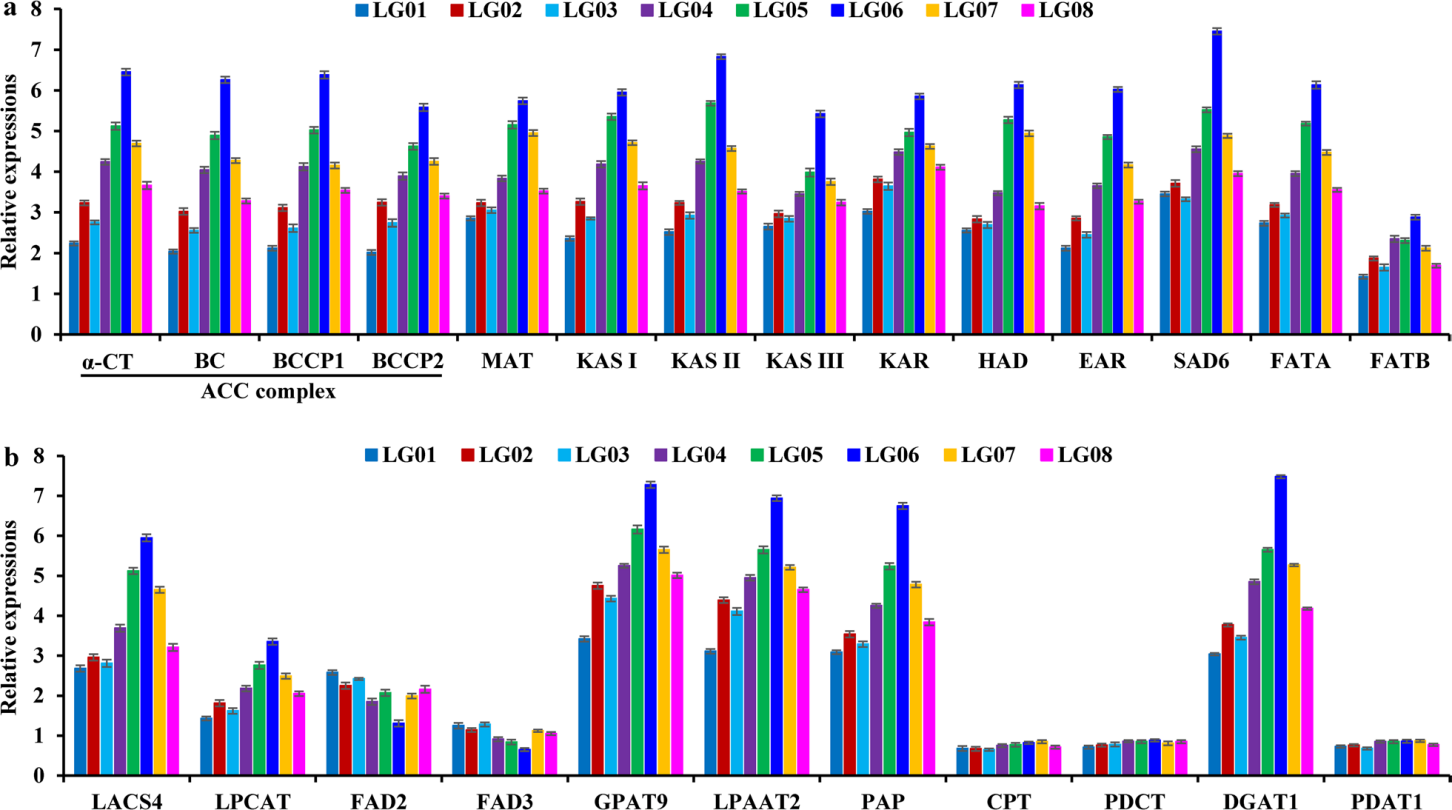
Analysis of transcript differences of enzymes for FA and TAG synthesis in *L. glauca* fruits of different accessions by qRT-PCR. **a** Transcript differences of enzymes for plastid FA synthesis in the fruits from different accessions. **b** Transcript differences of enzymes for TAG assembly in the fruits from different accessions. Both *RPL32e* and *UBC* genes were used as internal controls, and its expression level was arbitrarily set to 1.00 for standardization. Error bars are SD of three biological replicates with three technical repetitions each

Increasing FA and TAG biosynthesis and storage oil yield would expand economic value for oil plants [7]. Given differential transcripts of oil synthesis enzymes noted in developing *L. glauca* fruits by our recent transcriptome assay [12], it was necessary to explore quantitative relationship between the transcript levels of oil-synthesized genes and the amount of fruit oils from all accessions. By qRT-PCR, the abundantly coordinated transcripts were detected for FA synthetic enzymes, including acetyl-CoA carboxylase (ACC), malonyl-CoA-ACP transferase (MAT), fatty acyl-ACP thioesterase A/B (FATA/B), 3-ketoacyl ACP synthase I/II/III (KAS I/II/III), 3-ketoacyl ACP reductase (KAR), hydroxyacyl-ACP dehydrase (HAD), enoyl-ACP reductase (EAR) and 18:0-ACP desaturase 6 (SAD6) in the fruits of all accessions (Fig. 5a), which was the case for the enzymes for *de novo* TAG assembly [acyl-CoA:G3P acyltransferase 9 (GPAT9), acyl-CoA:LPA acyltransferase 2 (LPAAT2), acyl-CoA:DAG acyltransferase 1 (DGAT1) and PA phosphatase 2 (PAP2)] (Fig. 5b). A strong correlation of their transcript levels with fruit oil accumulation across all accessions (Figs. 1a, 5) emphasized their importance for FA synthesis and TAG assembly. Also of note was the role of FA exporter (FAX) in FA import into ER for TAG synthesis and long-chain acyl-CoA synthetase (LACS) in activating free FA to produce acyl-CoA pool [7, 27–29]. High transcript of FAX1/2 and LACS4 (Figs. 3c, 5b) was comparable to that of FA-synthesized enzymes (Figs. 5a), and showed a pattern that correlated with fruit oil content of all accessions (Fig. 1a), and thus referred that both FAX1/2 and LACS4 may be pivotal for FA export from plastid into ER for acyl-CoA pool generation destined to TAG assembly.

ER-located FAD2 and 3 is known for desaturation of C18:1 to produce C18:2 and then C18:3, respectively. Here, FAD2 showed a relative high transcript, but less transcript was marked for FAD3 (Fig. 5b), both of which were matched the level of C18:2 (high) and C18:3 (less) in fruit oils of all accessions (Table 1). High transcript similar to FAD2 was noted for acyl-CoA: lysophosphatidylcholine acyltransferase 2 (LPCAT2) (Fig. 5b), involved in the transfer of C18:1 into PC for desaturation and release of PUFA into acyl-CoA pool for TAG assembly [30], all of which displayed a strong correlation to the amount of C18:2 in fruit oils across all accessions (Fig. 1a), implying a specific contribution of FAD2 or LPCAT2 to PUFA production. Also, lower transcript was identified for PC: DAG cholinephosphotransferase (PDCT) and CDP-choline: DAG cholinephosphotransferase (CPT) (Fig. 5b), two enzymes for interconversion of DAG and PC to produce PUFA-rich TAG [30, 31], indicating its unimportance for TAG synthesis. Another was concerned for DAG acyltransferase 1 (PDAT1) for the last assembly of TAG [32], and its low transcript showed no substantial difference across different accessions (Fig. 5b), reflecting a very limited contribution of PDAT1 to TAG synthesis in the fruits of all accessions.

### Transcript differences in transcription factors for regulating fruit oil accumulation of different accessions

**Fig. 6:**
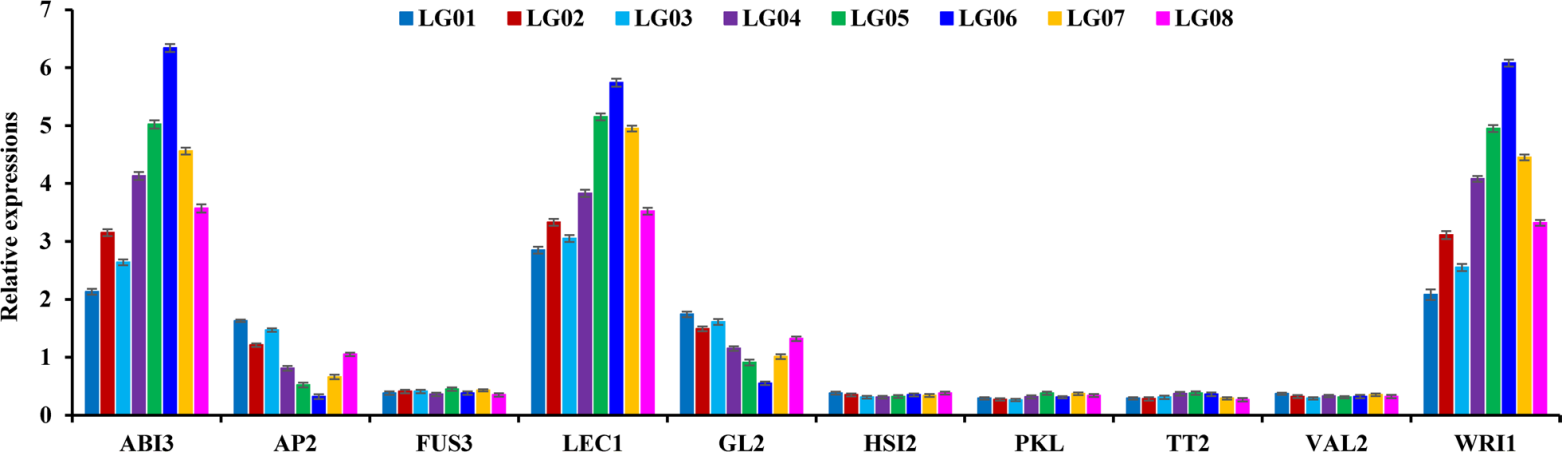
Transcript differences of transcription factors in *L. glauca* fruits of different accessions by qRT-PCR. Transcript differences of transcription factors (TFs) in the fruits from different accessions. Both *RPL32e* and *UBC* genes were used as the internal controls, and its expression level was arbitrarily set to 1.00 for standardization. Error bars are SD of three biological replicates with three technical repetitions each

**Fig. 7:**
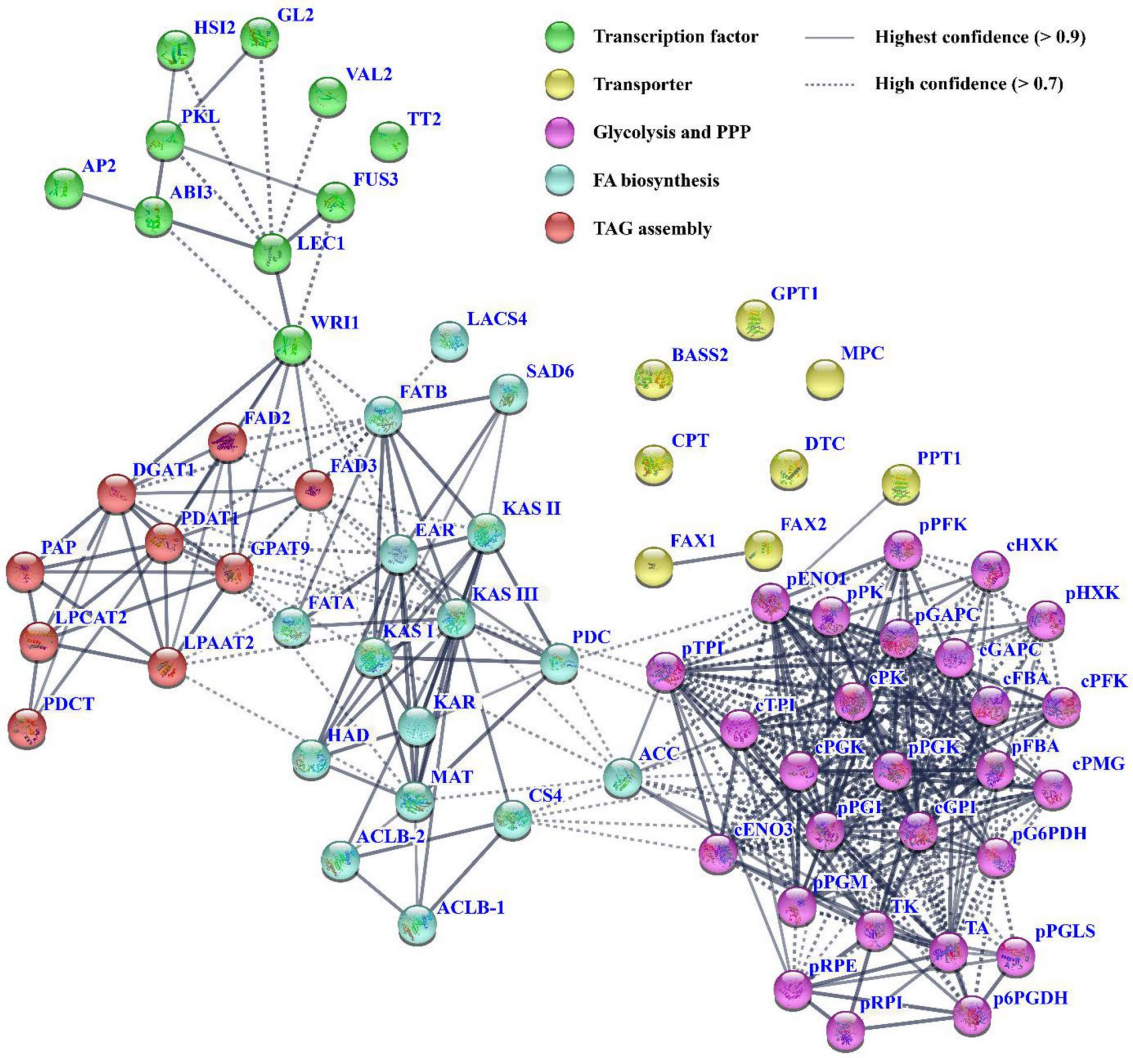
Analysis of protein interaction between transcription factors and oil-synthesized enzymes or specific transporters. A protein interaction network was constructed for transcription factors (TFs) and oil-synthesized enzymes or transporters using STRING software with high network connectivity (confidence score > 0.7)

Transcription factors (TFs) have shown to regulate oil biosynthesis of oil plants. Recently, differential transcripts were annotated for the TFs (ABI3, LEC1, WRI1, FUS3, AP2, GL2, HSI2, TT2, PKL and VAL2) in developing *L. glauca* fruits by our transcriptome analysis [12], which allowed us to explore the possible association of their transcript levels with accumulative amount of fruit oils across all accessions. Here, the transcripts of ABI3, LEC1 and WRI1 increased with the increase in fruit oil content of all accessions, while the transcripts of AP2 and GL2 showed a downtrend with the increase in fruit oil content, and less transcript was detected for TT2, FUS3, HSI2, VAL2 and PKL across all accessions (Figs. 1a, 6), reflecting a complex transcriptional regulation for fruit oil synthesis of different accessions. Also, we performed the protein interaction analysis for the above TFs and oil-synthesis enzymes as an attempt to highlight TF-mediated regulation mechanism for fruit oil biosynthesis, and found that the enzymes for oil accumulation (carbon source supply, FA synthesis and TAG assembly) were highly associated with WRI1, and LEC1 showed a strong interaction with WRI1 (Fig. 7), and thus considered that LEC1 and its targeted WRI1, located in the center position of interaction network, may contribute to regulate transcriptional expressions of enzymes relevant for fruit oil accumulation of all accessions.

## Discussion

### *L. glauca* fruit oil as novel promising woody feedstock for biodiesel production

In this work, to determine ideal germplasm for developing *L. glauca* fruit oils as woody biodiesel, a concurrent evaluation of oil content, FA composition, biodiesel yield, and fuel properties and predicted model construction was performed for *L. glauca* fruits of 8 selected accessions (Tables 1 and 2; Figs. 1, 2). Our results showed that the oil content (45.12–60.95%) of *L. glauca* fruits from different accessions (Fig. 1a) was higher than that of other traditional oil plants (11.68–40.28%) [7, 14, 24, 33–37], indicating that *L. glauca* accessions, especially high oil-bearing LG05 (53.16%), LG06 (60.95%) and LG07 (51.81%), had great advantage as biodiesel feedstock. Indeed, high yield of biodiesel from the fruit oils of LG05 (97.3%), LG06 (98.7%) and LG07 (96.8%) (Fig. 1b) could meet EN 14214 standard (96.5%), as was higher than that of *P. sibirica* (88.7%) [5, 15], emphasizing that the *L. glauca* fruit oils of these accessions (LG-05/06/07) may be suitable for producing high yield of biodiesel.

The ideal oils for high-quality biodiesel production should contain suitable amount of SFA, high percentage of MUFA and low proportion of PUFA [7, 15, 19, 38]. Here, all accession fruits contained high content of MUFA (52.43–78.46%) and low contents of PUFA (17.69–38.73%) and SFA (3.85–8.84%) (Fig. 1c), and notably, the amount of C18:1 and C18:2 in all accessions accounted for more than 84% of fruit oils (Table 1), indicating that the fruit oils from all accession may be as raw material suitable for biodiesel, coincided with previous results of several traditional woody oil plants [6, 7, 39–42]. Given that accession LG06 had the highest content of MUFA, the lowest levels of PUFA and SFA and a small ratio of C18:2/C18:1 (Fig. 1d–f), integrated with ideal value of IN (102.157), CN (51.15), OS (3.34 h), CP (− 9.91 °C) and CFPP (− 12.59 °C) in biodiesel (Table 2), revealing that the fruit oils from LG06 with idea FA compositions could meet the demand of high-quantity biodiesel production.

It was estimated that the total planting area of *L. glauca* was about 14,400 ha in China, and the average production of ripened fruits was about 11.5 ton /ha (equated to 5.0 kg of fruits per plant yield) [11, 12, 43]. Based on energy conservation principle and law of material invariance (including planting, fruit and biodiesel transport, oil and biodiesel production, and biodiesel combustion) for *P. chinensis* [44], the annual amount of biodiesel from *L. glauca* fruit oils was about 21,600 tons in China, and the resulting annual output value created by biodiesel was 178.84 million Yuan. Thus, it was concluded that our identified 3 accessions (LG05/LG06 /LG07) may be of interest for potential industrial application, specifically LG06 with the maximum yield of oil and biodiesel, and superior fuel properties.

### Strong funneling of carbon flux toward plastid acetyl-CoA for high FA production in fruits of all accessions

Several studies have shown that oil accumulation and FA composition vary considerably among different plants, provenances, tissues or developing stages [6, 7, 16, 17, 23, 24, 30, 33, 45–47], but the mechanism for controlling these differences still remains enigmatic. Thus, it is vital to unravel regulators for high-quality oil accumulation.

In oil plants, the source of PYR for high oil production is largely controlled by glycolysis in cytosol or plastid [12, 17, 23, 24, 46–50]. Here, transcript levels of glycolytic enzymes were much higher in plastid than in cytosol of all accessions (Fig. 3a, b), and notably, transcript abundance of plastid glycolytic enzymes was positively correlated with the increase of fruit oil content across all accessions (Figs. 1a, 3a), revealing a critical role of plastid glycolysis in providing PYR for FA synthesis, as also noted for several oil plants [7, 17, 23, 46, 50, 51]. In support of this finding, plastidic GPT1 (G6P transporter), PPT1 (PEP transporter) and BASS2 (PYR carrier) were transcriptionally coordinated with the increase of oil content in all accession fruits (Figs. 1a, 3c), implying a role of transporter-mediated import of G6P, PEP or PYR from cytosol into plastid glycolysis for FA synthesis in *L. glauca* fruits of all accessions, as could be evidenced by the fact that *PPT1* or *BASS2* overexpression promoted Arabidopsis seed oil increase [52, 53]. Yet, PPT1 transcript was on average 1.1- and 1.3-fold higher than that of GPT1 and BASS2, respectively (Fig. 3c), implying a major glycolytic carbon flux into plastid from cytosol at the level of PEP in *L. glauca* fruits, similar to other oil plants [17, 23, 26]. All these also clearly highlighted a strong coordinated funneling of glycolytic intermediate toward PYR in plastid, as could be further reflected by less transcript for mitochondrial MPC (PYR carrier) across all accessions (Fig. 3c). Aside from glycolysis, PPP as alternative pathway has shown to supply GAP for FA synthesis in oil plants [26, 51, 54]. Such pathway could be shown by our finding that the abundantly coordinated transcripts of plastid PPP and GPT1 displayed a close relation to the amount of fruit oils of all accessions (Figs. 1a, 3c, d), implying a portion of GPT1-mediated G6P import into plastid destined for FA synthesis. Yet, the fact that transcript level of plastid glycolytic enzymes was on average twofold higher than that of plastid PPP in all accessions (Fig. 3a, d) revealed that the necessity of carbon source for de novo FA synthesis in *L. glauca* fruits was mainly derived from plastidic glycolysis.

Acetyl-CoA, one vital precursor for de novo FA synthesis in plastid and its elongation in cytosol, was mostly derived from PYR via four alternative enzymes of PDC, ACL, ACS or CA [23, 55], all of which were shown with differential transcript in developing *L. glauca* fruits by our recent transcriptomic analysis [12]. Here, only plastidic PDC and cytosolic ACLB subunits (ACLB-1/-2) were highly expressed in all accessions (Fig. 4), while transcript level of plastidic PDC was on average fourfold higher than that of cytosolic ACLB across all accessions, and showed a positive association with the increased fruit oil of all accessions (Fig. 1a), emphasizing that the availability of acetyl-CoA for FA synthesis in *L. glauca* fruits of all accessions was derived from glycolytic PYR via the action of PDC in plastid, as also noted for other oilseed plants [24, 46]. Also noteworthy was the roles of cytosolic ACL and mitochondrial PDC in producing cytosolic acetyl-CoA for FA elongation, in which cytosolic PYR was imported into mitochondria by MPC (PYR transporter) for acetyl-CoA formation via mitochondrial PDC, as substrate for citrate formation by CS and then export by DTC transporter for cytosolic acetyl-CoA formation via ACL cleavage [7, 12, 23, 56]. Our observation that cytosolic ACLB-1/-2, and mitochondrial MPC, PDC and CS4 all showed low transcript (Figs. 3c, 4), coincided with small amount of C20:0 and C20:1 in fruit oils (Table 1), indicated that the production of cytosolic acetyl-CoA for FA elongation was coordinated at the transcript level.

Together, strongly increased plastid carbon supply via effective transport and glycolysis, and high action of plastid PDC, may provide great funneling of acetyl-CoA for high FA synthesis in *L. glauca* fruits of all accessions.

### FA flux channeled into de novo TAG assembly for high-quality oil accumulation in fruits of all accessions

The PUFA (C18:2 and C18:3) content is a key factor affecting the quality of plant oils for human health, biofuel and other purposes [31]. In oil plants, C18:1, which was produced from C18:0 by SAD6 in plastid, is desaturated to C18:2 and then C18:3 by ER-localized FAD2 and FAD3, respectively. Here, transcript level of SAD6 in the fruits across all accessions was on average 1.3- and 3.5-fold higher than that of FAD2 and FAD3, respectively, which closely matched the amount of C18:1, C18:2 and C18:3 in fruit oils of all accessions (Table 1 and Fig. 5b), and thus considered that C18:1 as the richest component in fruit oils may be mostly attributed to their differential coordinate transcripts. This was in line with previous results in different tissues of oil palm [57] and developing seeds of *P. sibirica* [58], and also be supported by high amount of C18:1 in *fad2*/*3* double mutant soybean [59].

The carbon chain length of FA and relative proportion of SFA and MUFA in the oils may be determined by de novo FA synthesis from acetyl-CoA in plastid [16]. Given a positive correlation between abundant transcript of FA-synthesized enzymes (ACC, MAT, KAR, HAD, KAS I/II/ III, EAR, FATA, FATB and SAD6) and accumulative amount of fruit oils across all accessions (Table 1 and Fig. 5a), it seems certain that the differences in fruit oil contents of different accessions may highly depend on the efficiency of FA synthesis in plastid, as also noted for oil palm [17]. Another difference across all accessions was transcript of ER-localized LACS4 crucial for activation of free FA to generate acyl-CoA pool for TAG assembly [16], and notably, its high transcript pattern (Fig. 5b) was closely related to fruit oil accumulative amount across all accessions (Table 1), indicating its importance in controlling plastid FA flux into acyl-CoA pool for TAG synthesis, as was evidenced by the increase of oil content in yeast mutant by overexpression of *B. napus LACS4* [29]. Considering that FAX1/2 as plastid transporter for export FA into ER responsible for TAG synthesis [7, 27, 28] was highly expressed in all accession fruits (Fig. 3c), it was believed that the abundantly coordinated transcripts of FA-synthesized enzymes, LACS4 and FAX1/2 may provide a strong channeling of plastid FA flux into ER to generate acyl-CoA pool destined for TAG synthesis.

Plant oil synthesis involves a complex metabolic network for TAG assembly with distinct FA profiles, and *de novo* assembly of TAG from G3P and acyl-CoA can produce oils with rich C18:1 [18, 30, 60]. Yet, how acyl flux is channeled to produce ideal oil composition in oil plants remains largely unclear. In this work, the accumulative amount of fruit oils with rich C18:1 was positively associated with high and increasing number of transcripts of crucial enzymes (GPAT9, LPAAT2 and PAP2) for de novo DAG synthesis via Kennedy pathway across all accessions (Table 1 and Fig. 5a), reflecting that direct utilization of de novo DAG for TAG synthesis may be as dominant route for *L. glauca* fruit oils with rich C18:1 of all accessions, as also noted in other oil plants [30, 61]. In general, C18:1 from plastid can be used directly for *de novo* DAG assembly by GPAT and LPAAT via Kennedy pathway, or incorporated into PC by LPCAT for desaturation by FAD2/3 [31]. Given that transcript level of LPCAT2 was on average 1.5-fold lower than that both GPAT9 and LPAAT2 for the first two acylation of TAG assembly in fruits of all accessions (Fig. 5), integrated with the fact of C18:1 as the richest component in fruit oils (Table 1), it seems certain that a major flux of C18:1 from acyl-CoA pool may be directly channeled into *de novo* DAG synthesis, and thus a small flux of C18:1 was used for desaturation. This conclusion could support earlier idea that LPCAT2 may transfer PC-derived PUFA directly into acyl-CoA pool for TAG synthesis via Kennedy pathway [62], and also was evidenced by the fact that *lpcat1*/*lpcat2* mutant reduced PUFA content in Arabidopsis seed oils [60], but *gpat9* mutant increased PUFA content and decreased seed oil content [63]. It was also noted that PUFA-rich TAG may be mostly attributed to PDCT or CPT [30, 31]. The fact that transcript levels of PDCT and CPT were on average 6.5-fold below that of both LPAAT and GPAT (Fig. 5), and showed no relation to variations for PUFA amount in fruit oils of all accessions (Fig. 5 and Table1), indicated a negligible role of PDCT or CPT in PUFA production in fruit oils of all accessions, also noted in developing *P. sibirica* seeds [8]. This was in line with previous notion of low PUFA production in oil palm caused by less transcript of PDCT and CPT [57], but contrasted with a major contribution of PC-derived DAG to TAG synthesis in developing seeds of Arabidopsis and soybean [31, 60, 64].

Also of note was DGAT and PDAT vital for the final acylation of TAG synthesis [32], but it remains unknown what extent both enzymes devote to the last TAG assembly in oil plants. Here, PDAT1, similar to both PDCT and CPT, showed less transcript and no difference across all accessions (Fig. 5b), it appeared that PDAT1 was not a key contributor for TAG synthesis in *L. glauca* fruits of all accessions. This corresponded to earlier results in oil plants from transcriptomic or metabolic analysis [17, 57, 65], and also confirmed by no effect of *PDAT1* mutant on seed oil content and FA profile [32]. In support of this conclusion, transcript level of DGAT1 was on average fivefold higher than that of PDAT1 across all accessions (Fig. 5b), and positively correlated with the amount of fruit oils (Table 1), revealing that DGAT1 may be a crucial regulator for oil synthesis in *L. glauca* fruits, coincided with metabolic and transcriptomic analyses of some oil plants [17, 24, 32, 46, 66], and also evidenced by the overexpression or mutant result of *DGAT1* in several oil plants [67–74].

**Fig. 8:**
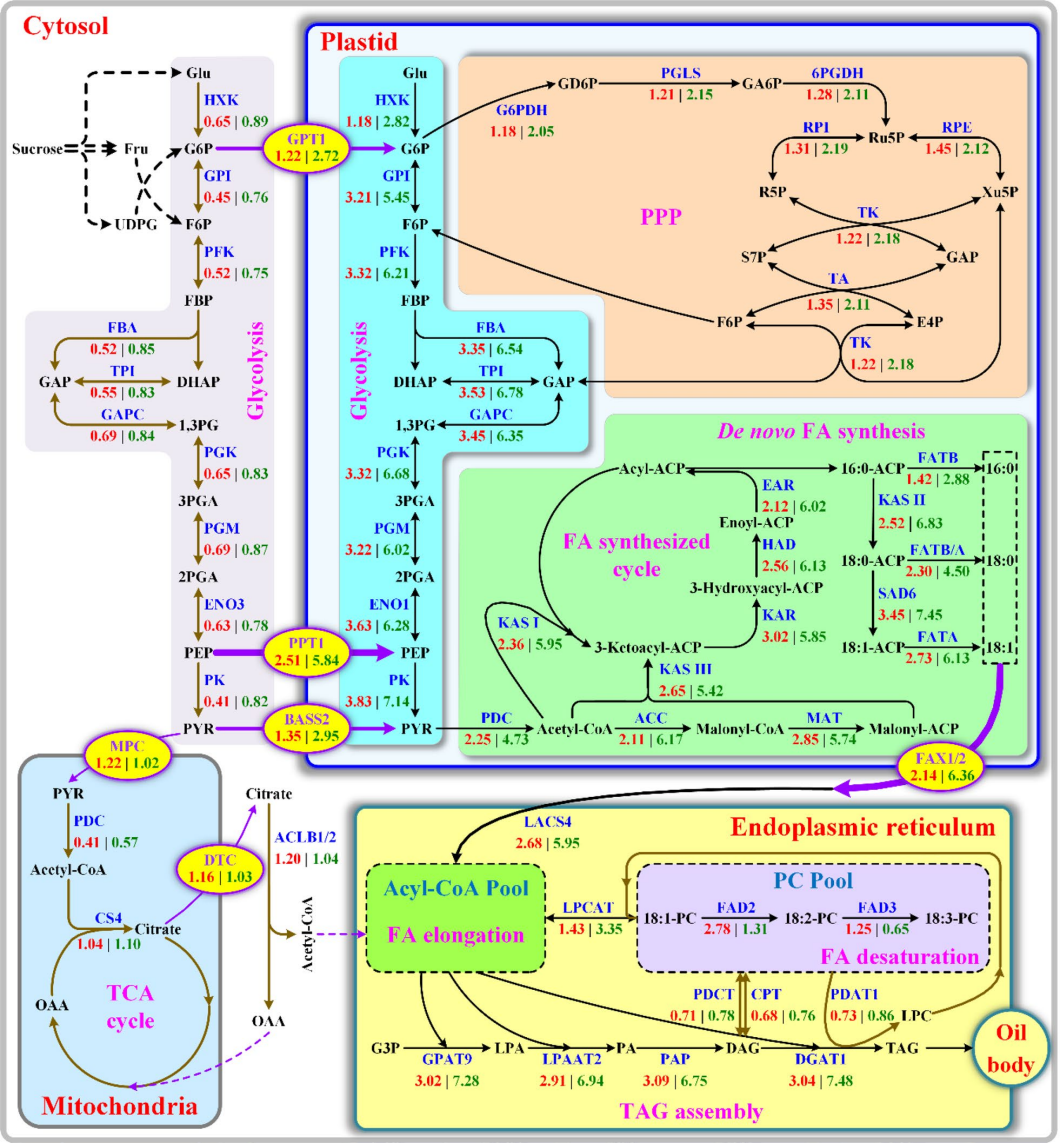
Characterization of central metabolic model for controlling carbon and FA flux for high oil synthesis in *L. glauca* fruits*.* The constructed metabolism regulatory network of carbon flux allocation for high-quality oil production in *L. glauca* fruits represents the most relevant pathways (glycolysis, PPP, acetyl-CoA formation, de novo FA synthesis and its elongation, plastidic FA export and desaturation, and TAG assembly) and includes all identified regulators (enzymes, transcription factors and transporters) with strong correlation with carbon flux partitioning into FA synthesis and TAG assembly, all of which are characterized by the comparative analysis of cross-accessions association of gene transcription level (qRT-PCR detection) with oil accumulative amount in fruits across all accessions, together with our recent transcriptome sequencing data from developing *L. glauca* fruits. All enzymes presented here are shown in blue. The values generated from qRT-PCR indicate relative transcript levels in the fruits from high-oil accession LG06 (green values) and low-oil accession LG01 (red values), all of which are calculated by the standardized set of expression level for inner reference gene to 1.00. The background color distinguishes different subcellular locations or pathways for FA and TAG synthesis as follows: gray and bright blue signify cytosolic and plastidic glycolysis, respectively; light red refers to plastidic PPP; light green signifies de novo FA synthesis; light blue signifies TCA cycle in mitochondria; yellow signifies TAG assembly in ER. *Black arrows* represent all reactions for major carbon allocation into FA synthesis and TAG assembly, and *brown arrows* represent minor carbon allocation for FA and TAG synthesis. *purple arrows* represent metabolite transport across intracellular membrane by specific transporters. Abbreviations for main enzymes, metabolites and transporters are as follows: ACC, acetyl-CoA carboxylase; BASS, pyruvate (PYR) carrier; DGAT, diacylglycerol (DAG) acyltransferase; EAR, enoyl-ACP reductase; ER, endoplasmic reticulum; FAD, FA desaturase; FATA/B, fatty acyl-ACP thioesterase A/B; FAX, FA exporter; G6PDH, glucose-6-phosphate (G6P) dehydrogenase; GPAT, acyl-CoA:G3P acyltransferase; GPI, G6P isomerase; GPT, G6P transporter; HAD, hydroxyacyl-ACP dehydrase; HXK, hexokinase; INV, invertase; KAR, ketoacyl-ACP reductase; KAS, 3-ketoacyl ACP synthase; LACS, long-chain acyl-CoA synthase; LPAAT, lysoPA acyltransferase; LPCAT, lysoPC acyltransferases; 6PGDH, 6-phosphogluconate dehydrogenase; PAP, phosphatidic acid (PA) phosphohydrolases; PC, phosphatidylcholine; PDAT, phospholipid:DAG acyltransferase; PDC, pyruvate (PYR) dehydrogenase complex; PK, PYR kinase; PPP, pentose phosphate pathway; PPT, PEP transporter; TAG, triacylglycerol; TPT, triose phosphate transporters

Overall, FAX1/2-mediated plastidic FA export, LACS4-regulated effective generation of acyl-CoA pool in ER, and LPCAT2-mediated C18:1 flux into PC for desaturation and PUFA back into acyl-CoA pool, integrated with the abundantly coordinated transcripts for the enzymes of FA synthesis and de novo TAG assembly via Kennedy pathway, was crucial for the eventual high-quality oil accumulation in *L. glauca* fruits of all accessions (Fig. 8).

### LEC1/WRI1-mediated regulatory network for oil accumulation in *L. glauca* fruits of all accessions

Plant FA and TAG biosynthesis, involving a series of gene expression, is highly regulated by several TFs [75–82]. Our findings that high transcript patterns of ABI3, LEC1 and WRI1 were positively correlated with the amount of fruit oils across all accessions, but the down-regulated profiles of GL2 and AP2 showed an inverse relationship with oil content (Figs. 1a, 6), indicated that ABI3, WRI1 and LEC1 may act as positive regulators for gene expression relevant for fruit oil synthesis of all accessions, but AP2 and GL2 may play negative regulatory factors, as was noted for developing *P. sibirica* seeds [8], and also compatible with our recent transcriptome result of developing *L. glauca* fruits [12]. Of note, WRI1, has been shown to control the expressions of its targeted genes involved in glycolysis, and FA and TAG biosynthesis during oilseed development [82–84]. This could be directly shown by the fact that the enzymes for carbon allocation (plastid glycolysis and acetyl-CoA formation), de novo FA synthesis and TAG assembly were transcriptionally coordinated with WRI1 across all accession fruits (Figs. 5, 6), which in turn was closely related to the fruit oil content (Fig. 1a). Such strong relationship implied that these genes may be as the targets were activated by WRI1, in which carbon flux could be efficiently channeled into oil synthetic pathway for oil accumulation in the fruits of all accessions by collaborative manner, similar to the results of other oilseed plants [57, 84–86], and also evidenced by the increase in FA synthesis and oil accumulation by ectopic expression of *WRI1* from many oilseed plants [75–77, 80, 81, 84–86]. WRI1 is known to be up-regulated by LEC1/2, ABI3 or FUS3 [85]. A similar profile of high transcript for ABI3, LEC1 and WRI1 in all accessions (Fig. 5) revealed *WRI1* as a target gene of LEC1 or ABI3, as was also reported in *A. thaliana* and *P. sibirica* seeds [24, 87], but contrasted with the idea that WRI1 function might be independent of upstream TFs in controlling oil biosynthesis in oil palm [17]. LEC1 has been identified as a central regulator of oil synthesis in developing seeds [83, 88], and overexpression of *LEC1* increased oil content by up-regulating several genes relevant for FA and oil synthesis in oilseed plants [78, 83, 89]. Overall, the abundantly coordinated transcripts were identified for the TFs (LEC1 and WRI1) and the enzymes of carbon flux allocation (plastidic glycolysis and acetyl-CoA generation) and FA synthesis as well as TAG assembly (Figs. 4–6), showing a positive correlation with the accumulative amount of fruits oils across all accessions (Table 1), and thus revealed that LEC1/WRI1-mediated transcription regulatory network may play a major role in FA synthesis and oil accumulation in *L. glauca* fruits of different accessions (Fig. 7).

## Conclusions

In this work, the cross-accessions comparisons were conducted on oil content, FA profile, biodiesel yield, fuel property and prediction model construction to determine superior accession (LG06) as promising feedstock for ideal biodiesel production. An integrated assay for the associations between oil-synthesized gene transcription level and oil accumulative amount was used to identify some key regulators (enzymes, transcription factors and transporters) required for high-quality oil accumulation of *L. glauca* fruits, including carbon allocation (plastidic glycolysis and PPP), metabolite transporter (plastid GPT1/PPT1/BASS2/FAX1/2), acetyl-CoA formation (plastidic PDH), transcription factor (LEC1/WRI1), FA synthesis (ACC, MAT, FATA/B, KAS I/II/III, KAR, HAD, EAR and SAD6), and TAG assembly (LACS4, LPCAT2, FAD2/3, GPAT9, LPAAT2, PAP and DGAT1). Such exploration has led to central metabolic model established for controlling carbon and FA flux for high oil synthesis in *L. glauca* fruits (Fig. 8)***.*** Together, our findings could facilitate development of *L. glauca* fruit oils as biodiesel feedstock, and provide new insight into regulatory mechanism of high oil production destined for further metabolic engineering of oil accumulation in *L. glauca* and other oil plants.

## Materials and methods

### Collection of fruit samples

A total of 8 plus trees (defined as superior trees) of *L. glauca* (germplasm accessions LG01, LG02, LG03, LG04, LG05, LG06, LG07 and LG08) with high fruit yield were selected from different regions located in south of the Qinling Mountains (geographical coordinates approximately E113°00′-119°26′, N28°26′-33°49′), and planted in JiGong Mountain National Natural Reserve (E114°06′, N32°125′) of Henan Province in China. The ripened fresh fruits were collected from 10 year-old tree, and 150 fruits from 10 trees (15 fruits each tree) of each accession were selected each time. All samples were stored at − 80 °C until use.

### Oil extraction and trans-esterification

About 50 g of fresh fruit (3 samples per accession) were crushed into power, and then the oils were extracted with petroleum ether using Soxhlet apparatus at 45–50 °C [12]. After extraction for 6–8 h, the oil was separated from organic mixture by rotary evaporator (LABORTA 4000-efficient, Germany), and then dried at 105 °C in the ventilated drying oven. The content of extracted oil from each accession fruit was calculated as the difference between the weights of fruit sample before and after extraction, and expressed as the percentage of the extracted oil weight to fresh fruit weight (%, g/g). To analyze FA compositions, the fruit oils from each accession were *trans*-esterified as previously described [90]. All analysis was conducted on triplicate.

### Analyses of FAMEs and biodiesel yield

The FA methyl esters (FAMEs) obtained from each accession fruit was used to determine FA compositions using Agilent 6890 (California, USA) gas chromatograph equipped with flame ionization detector (GC-FID) [12]. The HP-INNOWax capillary column (inner diameter 0.32 mm, film thickness 0.5 μm, split 1:20) was used, and the temperature was programmed at 60 °C, with a rise of 4 °C min^−1^ to 220 °C and heated to 240 °C for 10 min. The carrier gas was helium with a flow rate of 1.0 mL min^−1^. The peaks of FAMEs were identified by comparing their retention time with that of the known standards, and peak integration was performed by applying HP3398A software. Each FAME assay was performed in triplicate, and the data were present as the mean. The biodiesel yield was calculated by the previous method [7, 12], where the yield was expressed as the percentage (%, g/g) of the obtained total amount of FAMEs (g) to the used amount of raw oils (g).

### Quality of biodiesel fuel property

The fuel properties (IV, CN, CP, CFPP, OS, D and KV) were calculated from the FAME compositions of fruit oils by our previous methods [7, 12]. Also, two additional vital parameters based on the type of FAs were defined for biodiesel feedstock: degree of unsaturation (DU) of FAs and chain length saturated factor (LCSF), of which LCSF is calculated taking into account the compositions of saturated FAs and their corresponding melting point, and DU value is calculated from the amount of monounsaturated and polyunsaturated FAs (%) in fruit oils according to the following equations [7, 12, 14, 15]. All assay was performed for triplicate, and biodiesel fuel properties of fruit oils were compared with the relevant standards of EN 14214 (European), ASTM D6751 (USA) and GB/T 20828 (China):

$${\text{DU}} = { 1} \times \, [{\text{Cn}}:1\left( {{\text{wt}}.\% } \right)] \, + { 2} \times \left[ {{\text{Cn}}:{2 }\left( {{\text{wt}}.\% } \right)} \right] + { 3} \times \left[ {{\text{Cn}}:{3 }\left( {{\text{wt}}.\% } \right)} \right],$$

$${\text{LCSF}} = \sum \,({\text{MPn}} \times {\text{Cn}})/{1}00,$$

where Cn is percentage of the *n*th saturated FAs and MPn is the melting point (MP) of *n*th saturated FAs.

### Construction of prediction model for biodiesel properties of raw fruit oils

Triangular prediction model of raw fruit oils from different accessions was constructed based on the influence of FA compositions [7, 12]. To predict biodiesel properties of fruit oils from each accession, the percentages of SFA, MUFA and PUFA from each accession fruit were calculated to outline triangular prediction graph, in which three angular points of the triangle meant the 100% of SFA, MUFA and PUFA, respectively. In triangular graph, the region existed at the far end of the polyunsaturated angular point (lower left vertex) and the saturated angular point (lower right vertex) was delineated to predict the biodiesel fuel properties, taking into account the CN, IV, CFPP and OS [7, 12].

### qRT-PCR analysis

Total RNA was extracted using RNeasy Plant Mini Kits (Qiagen, Inc., USA), and the obtained RNA was qualified and quantified using Nanodrop ND-1000 Spectrophotometer. All the samples showed a 260/280 nm ratio from 1.9 to 2.1, and then was reverse transcribed using the Reverse Transcription System (Promega). All the qRT-PCR primers (Additional file 1: Table S1) were designed by our recent transcriptome results of *L. glauca* fruits [12] using PrimerQuest (http://www.idtdna.com/PrimerQuest/Home/Index). The genes encoding large subunit ribosomal protein L32e (RPL32e) and ubiquitin-conjugating enzyme (UBC) were used as inner references [12, 90]. The qRT-PCR was conducted on 7500 Real-Time PCR System by SYBR Premix Ex Taq Kit (TaKaRa). The negative controls consisting of nuclease-free water instead of template, and reverse transcriptase controls prepared by substituting reverse transcriptase for nuclease-free water in cDNA synthesis step were included in all analyses for each primer pair. Three biological replicates with three technical repetitions each were performed for qRT-PCR.

### Protein interaction network analysis

The Search Tool for the Retrieval of Interacting Genes/Proteins (STRING version 9.0, http://string90.embl.de/) was employed to analyze the potential interactions between all our identified TFs and oil-synthesized enzymes or specific transporters. STRING analysis was conducted using high confidence (score 0.7), and cluster analysis was performed by using *k*-means with a value of *k* = 3.

## Supplementary Information

**Additional file 1: Table S1.** The information of all primers used in this study for qRT-PCR analysis.

## Data Availability

All data generated or analyzed during this study are included in this published article and its Additional files.

## Abbreviations

ABI3: Abscisic acid insensitive3
ACC: Acetyl-CoA carboxylase
ACLA/B: ATP-citrate lyase subunit A/B
BASS: Pyruvate (PYR) carrier (bile acid: sodium symporter)
CFPP: Cold filter plugging point
CN: Cetane number
CP: Cloud point
CPT: CDP-choline:DAG cholinephosphotransferase
DGAT: Diacylglycerol (DAG) acyltransferase
DTC: Dicarboxylate/tricarboxylate carrier
EAR: Enoyl-ACP reductase
ENO: Enolase
ER: Endoplasmic reticulum
FAD: Fatty acid (FA) desaturase
FAMEs: FA methyl esters
FATA: Fatty acyl-ACP thioesterase A
FATB: Fatty acyl-ACP thioesterase B
FAX: FA exporter
FBA: Fructose-bisphosphate aldolase
FK: Fructokinase
FUS3: FUSCA3
G6PDH: Glucose-6-phosphate (G6P) dehydrogenase
GAPA: Glyceraldehyde 3-phosphate (GAP) dehydrogenase subunit A
GLT: Glycolipid transporter
GPAT: Acyl-CoA:G3P acyltransferase
GPI: G6P isomerase
GPT: G6P transporter
HAD: Hydroxyacyl-ACP dehydrase
HXK: Hexokinase
INV: Invertase
KAR: Ketoacyl-ACP reductase
KAS: 3-Ketoacyl ACP synthase
LACS: Long-chain acyl-CoA synthase
LEC1: Leafy cotyledon1
LPAAT: LysoPA acyltransferase
LPCAT: LysoPC acyltransferases
MAT: Malonyl-CoA-ACP transferase
MPC: PYR carrier
6PGDH: 6-Phosphogluconate dehydrogenase
PAP: Phosphatidic acid (PA) phosphohydrolases
PDAT: Phospholipid:DAG acyltransferase
PDC: Pyruvate (PYR) dehydrogenase complex
PDCT: PC:DAG cholinephosphotransferase
PFK: Phosphofructokinase
PFP: Pyrophosphate phosphofructokinase
PGK: Phosphoglycerate kinase
PGLS: 6-Phosphogluconolactonase
PGM: Phosphoglycerate mutase
PK: PYR kinase
PPP: Pentose phosphate pathway
PPT: PEP transporter
qRT-PCR: Quantitative real time RT-PCR
RPE: Ribulose-5-phosphate (Ru5P) epimerase
RPI: Ribose 5-phosphate isomerase
TA: Transaldolase
TAG: Triacylglycerol
TK: Transketolase
TPI: Triose phosphate isomerase
TPT: Triose phosphate transporters
WRI1: Wrinkled1
UGPG: UDP-glucose
